# Erythropoietin Reduces Inflammation, Oxidative Stress, and Apoptosis in a Rat Model of Bleomycin-Induced Idiopathic Pulmonary Fibrosis

**DOI:** 10.3390/jpm14090972

**Published:** 2024-09-13

**Authors:** Drosos Tsavlis, Kalliopi Domvri, Konstantinos Porpodis, Stamatia Papoutsopoulou, Doxakis Anestakis, Anna Tzoumaka, Soultana Meditskou, Konstantina Symeonidoy, Evangelia Spandou

**Affiliations:** 1 Laboratory of Physiology, School of Medicine, Aristotle University of Thessaloniki, 54124 Thessaloniki, Greece; atzoumaka@auth.gr (A.T.); ksymeoni@auth.gr (K.S.); espandou@auth.gr (E.S.); 2 Laboratory of Histology-Embryology, School of Medicine, Aristotle University of Thessaloniki, 54124 Thessaloniki, Greece; kdomvrid@auth.gr (K.D.); sefthym@auth.gr (S.M.); 3 Department of Pulmonology, Aristotle University of Thessaloniki, General Hospital G. Papanikolaou, 57010 Thessaloniki, Greece; kporpodis@auth.gr; 4 Department of Biochemistry and Biotechnology, Faculty of Life Sciences, University of Thessaly, Mezourlo, 41500 Larissa, Greece; stapapou@uth.gr; 5 Department of Pathology & Forensic Sciences, Medical School, University of Cyprus, 1678 Nicosia, Cyprus; anestakis.doxakis@ucy.ac.cy

**Keywords:** idiopathic pulmonary fibrosis, erythropoietin, bleomycin, anti-inflammatory, antioxidant, antiapoptotic

## Abstract

Background: Idiopathic pulmonary fibrosis (IPF) is a lethal interstitial disease with unknown etiology and no effective cure, posing a great health burden to society. Erythropoietin (EPO) has been demonstrated to have protective roles in various tissues such as brain, spinal cord, heart, kidney and lung tissues. In this study, we investigate the specific anti-inflammatory, antioxidant and antiapoptotic effects of erythropoietin on lung tissue in a bleomycin-induced rat model of idiopathic pulmonary fibrosis. Methods: Recombinant human EPO or saline was injected, and the animals were monitored for 14 days after bleomycin instillation. Their hematocrit and serum EPO levels were determined. Histological and immunohistochemical analyses were performed. Results: The extent of tissue injury, determined through morphometric analysis, was significantly decreased in size in animals treated with erythropoietin. An immunohistochemical analysis of the expression of cyclooxygenase-2 (COX-2), inducible synthase of nitric oxide (i-NOS), metalloproteinase-9 (MMP-9), erythropoietin receptor (EPO-R), and cytochrome-C (cyt-C) found these enzymes to be decreased in a statistically significant manner in animals treated with erythropoietin when compared to a non-treated group. Conclusions: The reduced expression of COX-2, i-NOS, MMP-9, EPO-R, and i-NOS in the lung tissues of animals treated with EPO indicates the anti-inflammatory, antioxidant and antiapoptotic action of erythropoietin, suggesting its potential therapeutic role in pulmonary fibrosis.

## 1. Introduction

Idiopathic pulmonary fibrosis (IPF) is a progressive and ultimately fatal disorder of unknown etiology characterized by interstitial fibrosis of the lungs, where the prognosis without treatment is 2–3 years [1]. Although the Food and Drug Administration approved nintedanib and pirfenidone for the treatment of IPF in 2014, leading to a better prognosis by slowing down the progression of the disease, IPF is still incurable due to its complexity and not well-understood pathogenesis [2].

Despite evidence that inflammation plays an important role in the pathogenesis of IPF, it has been suggested that the inflammatory changes seen in IPF may occur independently of the primary fibrotic remodeling process [3]. Therefore, a “one size fits all” approach regarding the role of inflammation in IPF does not apply. It is essential to develop a personalized treatment approach, based on genomics or biomarkers and inflammatory profiles, for the selection of IPF patients who may be eligible for co-treatment with anti-inflammatory therapies besides pirfenidone and nintedanib, now considered the standard of care in “anti-fibrotic” medication [4].

In the last 20 years, erythropoietin (EPO)—the main erythropoietic hormone—has emerged as an important cytoprotective cytokine in several tissues, including the brain, heart, spinal cord, kidneys and lungs, in animal models of pulmonary diseases [5,6,7,8,9,10]. Many studies have demonstrated erythropoietin’s anti-inflammatory action in the lungs, suggesting that it could be a potential candidate for delaying the development of the inflammatory environment and its progression to fibrosis [5,11,12,13]. Researchers have focused their interest on the characteristic reduction or partial inactivation of inflammatory factors following systemic administration of erythropoietin to experimental animals. It has also been reported that EPO protects various tissues through antiapoptotic effects and that it can reduce glucose-induced oxidative stress through acting as a direct antioxidant [14,15]. Additionally, EPO administration reduced the levels of proinflammatory cytokines in the circulation, preserved microvascular endothelial cell integrity, and reduced oxidative stress-associated lipid peroxidation [16]. It is obvious that, due to EPO’s multiple properties, further research is needed to fully elucidate the underlying mechanisms of its protective action in pulmonary fibrosis, as well as to clarify whether it might be a powerful solution against this fatal disease in the future. No published research has studied the impact of EPO on the morbidity or mortality of patients with pulmonary fibrosis, and very little information is known in the context of other lung diseases such as chronic obstructive pulmonary disease [17]. However, there are numerous publications in the literature on the positive effects of EPO on the morbidity and mortality of renal or heart patients [18,19,20]. The aim of the present study was to contribute to this field through the assessment of specific biomarkers, such as cyclooxygenase (COX-2), inducible nitric oxide synthase (i-NOS), metalloproteinase-9 (MMP-9), erythropoietin receptor (EPO-R), and cytochrome C (Cyt-C), in a bleomycin-induced model of pulmonary fibrosis. To the best of our knowledge, this is the first study to investigate EPO’s whole protective role, regarding its anti-inflammatory, antioxidant, and antiapoptotic effects simultaneously, in a bleomycin-induced IPF model through assessing specific biomarkers. These biomarkers are strongly expressed in inflammatory and fibrotic processes, including uncontrolled degradation of the matrix (COX-2, MMP-9); in highly oxidizing environments with increased production of oxygen free radicals (i-NOS); or in conditions of cell death and apoptosis (EPO-R, cyt-C). Furthermore, according to the literature, the bleomycin-induced fibrosis model is the most widely used and well-established animal model of pulmonary fibrosis [21]), and it has provided valuable insights into the process of the disease—for example, enabling elucidation of the importance of the role of TGF-β in the development of IPF —and has also been used in the pre-clinical development of nintedanib [22]. 

## 2. Materials and Methods

### 2.1. Chemicals/Reagents

Bleomycin hydrochloride solution (TV3155-01, Teva, Basel, Switzerland) was purchased from Nippon Kayakou, Tokyo, Japan (15 mg/0.4 mL). The concentration of the administered solution was determined at 7.5 mg/kg of animal body weight. The formulation of EPO used was from Eprex^®^ Company (Nova Scotia Health, Halifax, NS, Canada), and the administered dose was 2000 IU/kg of animal body weight.

### 2.2. Animals and Experimental Design

A total of 50 weaned male Wistar rats were used, with a body weight of 200–350 gr. Rats were obtained from the Veterinary Medicine School of Aristotle University of Thessaloniki, maintained in a controlled environment with a 12 h light/12 h dark cycle (lights on at 08:00/lights off at 20:00), at a standard temperature of 22 ± 2 °C, and had unrestricted access to food and water. 

Animals were divided into five groups (*n* = 10/group), as follows: Control group 1 (CNT), no formulation was injected into the animals; Control group 2 (CNT SAL), saline solution (NaCl 0.9%) was injected both intratracheally and intraperitoneally into the animals; Control group 3 (CNT SAL/EPO), saline solution (NaCl 0.9%) and EPO were injected intraperitoneally into the animals; Bleomycin group (BLM/SAL), bleomycin was injected intratracheally into the animals; and EPO group (BLM/EPO).

On the first day of the experiment, the animals were anesthetized via intraperitoneal administration of 4.5% (1 mL/kg) of chloral hydrate. The trachea was exposed via a midline neck incision, a catheter was inserted into the trachea, and using a sterile 26G syringe, saline (for Groups 2 and 5) or bleomycin (for Groups 3 and 4) was slowly injected in the trachea. The low injection rate was intended to avoid bradycardia, cardiac flexion, pulmonary edema, and the experimental animal’s death. Once endotracheal injection was complete, the surgical incision was sutured with silk thread (Mersilk 0/2). After the end of the experiment, saline (Group 2) and erythropoietin (Groups 4 and 5) was administered intraperitoneally. At the end of these manipulations, all animals were placed in their individual cages, where they remained for 14 days, and were monitored daily for any change in their behavior and activity.

### 2.3. Blood Sampling—Hematocrit Measurement

Blood collection from the jugular vein was performed twice for each experimental animal, on Day 1 and Day 14 of the experiment. Each blood sample was measured twice, in order to determine the hematocrit, with the micro hemacytometer measurement device. The remainder was transferred to 1 mL centrifuge tubes (Eppendorf) and was left to coagulate before performing centrifugation at 3500 rpm for 15 min for serum uptake. Serum samples were frozen and maintained at −20 °C until measurement of EPO levels via chemiluminescence.

### 2.4. Measurement of EPO Levels in Blood Serum

Serum levels of EPO were measured using the chemiluminescence method in an IMMULITE 2000 analyzer from Siemens^®^ (Siemens, Munich, Germany) [23]. The EPO levels in the blood serum of the experimental animals of all groups were measured on both the 1st and the 14th day of the experimental procedure.

### 2.5. Histochemical Staining of Hematoxylin–Eosin (H-E)

Hematoxylin–eosin staining was used in histological preparations to identify inflammation and fibrosis areas, evaluated using the Image-Pro Plus Software Version 4.1 (USA) for morphometric analysis. Pulmonary damage was assessed through staging the damage severity using a semi-quantitative scale with 3 grades from 0 to 2: 0 → presence of <25% of the surface of the histological preparation, 1 → presence of 25–50% of the surface area of the histological preparation, 2 → presence of >50% of the surface of the histological preparation [24,25].

### 2.6. Immunohistochemical Assessments

Immunohistochemical staining for cyclooxygenase (COX-2, for inflammation), inducible nitric oxide synthase (i-NOS, for oxidative stress), metalloproteinase-9 (MMP-9, for inflammation and apoptosis), erythropoietin receptor (EPO-R, for apoptosis) and cytochrome C (Cyt-C, for apoptosis) were performed. The antibodies included in the immunohistochemical study were as follows: monoclonal anti-rabbit antibody against cyclooxygenase-2 (COX-2; 1:50, Spring Biosciences, Pleasanton, CA, USA), polyclonal anti-rabbit antibody against inducible nitric oxide synthase (i-NOS; 1:100, Chemicon International, Temecula, CA, USA), polyclonal anti-goat antibody against metalloproteinase-9 (MMP-9; 1:50, Santa Cruz Biotechnology, Dallas, TX, USA), rabbit monoclonal anti-rabbit antibody against the erythropoietin receptor (EPO-R; 1:50, Santa Cruz Biotechnology), and polyclonal anti-goat antibody against cytochrome C (Cyt-C; 1: 100, Santa Cruz Biotechnology).

Evaluation of protein expression was carried out by combining the intensity of the staining indicating the positive cells in the studied preparations and the extent to which they covered them in the test preparation. The staining intensity in the histological samples was scored on a scale from 0 to 3, where 0 was characterized as no expression of the antibody, 1 was characterized as mild expression, 2 was characterized as moderate expression, and 3 was characterized as the strongest expression in the test sample. The extent covered by immunohistochemical staining in histological preparations was also classified into four corresponding categories based on the mucosal percentage of each sample in which the test protein was observed. The final result for each preparation was the product of the extent of the positive (for each antibody) cells on the intensity of the antibody-related staining (Soslow method), and, based on this product, the final classification of the expression of antibodies in a percentage range of four categories was obtained as follows: 0 → expression at 0–25%, 1 → expression at 25–50%, 2 → expression at 50–75%, 3 → expression at 75–100% [24].

### 2.7. Statistical Analysis

Statistical analysis was performed using the SPSS software (version 21.0 IBM SPSS statistical software, Armonk, NY, USA). Descriptive statistics were calculated, and quantitative data are summarized as mean and standard deviation (SD). The correlation parameters were obtained using the Pearson correlation coefficient (r). The differences between groups concerning body weight, hematocrit, and erythropoietin measurements were determined with two-factor repeated-measures ANOVA, and a paired *t*-test was used for each group for the differences between the 1st and 14 days of the experiment. The level of significance considered for all statistical analyses was set at *p* < 0.001.

## 3. Results

### 3.1. Bodyweight, Hematocrit, and Erythropoietin Measurements

The body weight, hematocrit, and EPO results for all experimental groups at day 1 and day 14 are presented in Table 1. A significant difference was observed in the body weight between all groups on the 14th day (*p* < 0.001). In the BLM group, body weight was remarkably decreased, presenting the lowest value (227.25 g). Regarding the hematocrit and EPO levels, no statistically significant differences were found for each group between the 1st and the 14th day of the experiment (*p* > 0.05).

### 3.2. Histological Examination

**Figure 1:**
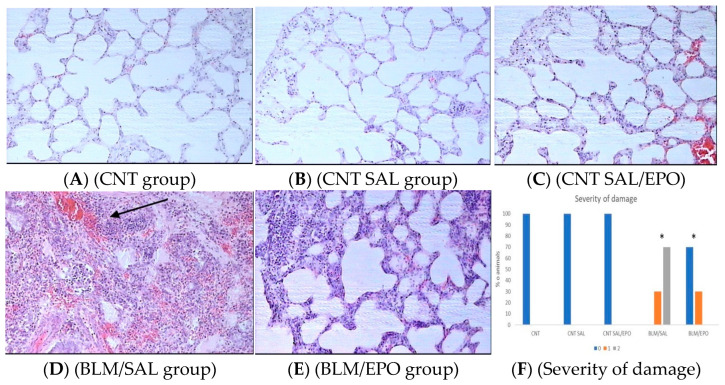
(**A**) Normal pulmonary parenchyma, absence of inflammation and fibrosis; (**B**) physiological architecture of alveoli and interstitial space; (**C**) milder pneumonitis; (**D**) massive accumulation of macrophages and diffuse bleeding (arrow); (**E**) fibrosis retreating and ventilation restoration; (**F**) severity of damage as a percentage (%) of the experimental animals per study group, classified on a 0–2 scale. * *p* < 0.001, with statistically significant differences between BLM/SAL and BLM/EPO groups.

Pulmonary lesions were assessed through histochemical staining (using hematoxylin–eosin) and morphometric analysis. In particular, no significant lesions of the pulmonary parenchyma and alveolar space were observed in CNT (Figure 1A). In the CNT SAL and CNT SAL/EPO groups (Figure 1B,C), apart from very mild pneumonitis—as expected after the endotracheal saline—no other damage was observed, and histological preparations were similar to those in the CNT group. In these two groups, the architectural structure remained stable, with a few leukocytes observed in the interstitial space but, in most cases, no fibrotic elements such as fibroblasts and collagen. In the BLM/SAL group, the inflammatory and fibrotic lesions were very pronounced. The medial connective tissue had intense infiltration by lymphocytes and fibroblasts (Figure 1D). Extensive areas of the alveolar wall exhibited large areas of inflammatory tissue. In the BLM/EPO group, lesions of the pulmonary parenchyma and alveolar space were limited compared to the BLM/SAL group (Figure 1E). Distinguished significant improvements, manifested as remarkable reductions in the fibrotic area’s surface and intensity of fibrotic effects (e.g., the accumulation of lymphocytes, macrophages, and fibroblasts), were also observed. There were areas with altered fibrous and inflammatory elements in the BLM/SAL group that did not indicate warm and intense inflammation or fibrosis but, instead, a much-improved condition.

The severity of the damage, depending on its location and extent, was assessed using a semi-quantitative scale of 3 classes from 0 to 2, as shown in Figure 1. More specifically, in the control groups (CNT, CNT SAL, and CNT SAL/EPO), no severity of damage was observed (as expected), whereas in the BLM/SAL group, class I was found at 30% and class II at 70%. In contrast, in the BLM/EPO group, class II was found at 0% and class I at 30%. Furthermore, statistically significant differences were found between the BLM/SAL and BLM/EPO groups, demonstrating lower severity of damage in the BLM/EPO group than in the BLM/SAL group (*p* < 0.001).

### 3.3. Immunohistochemical Examination

**Figure 2:**
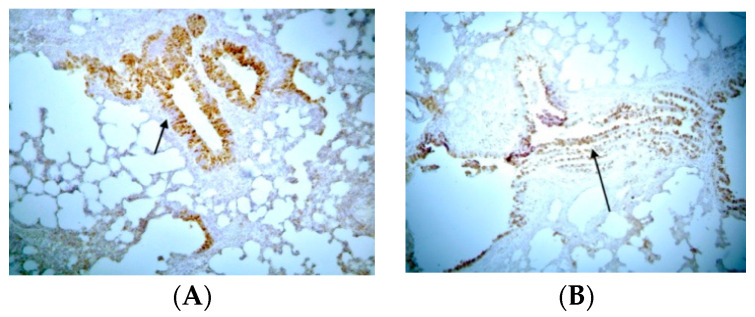
Representative data of COX-2 expression: (**A**) BLM/SAL group, diffuse and strongly positive expression of the enzyme (arrow); (**B**) BLM/EPO group, less positive staining of the substrate with intense staining of the bronchus’ smooth muscle fibers (arrow).

**Figure 3:**
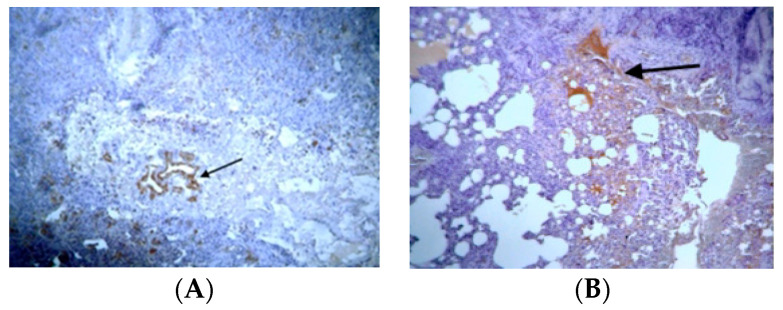
Representative data of i-NOS expression: (**A**) BLM/SAL group, intense positive cells in the bronchial epithelium (arrow); (**B**) BLM/EPO group, resurfacing healthy cells and small foci of positive cells in the interstitial space (arrow).

**Figure 4:**
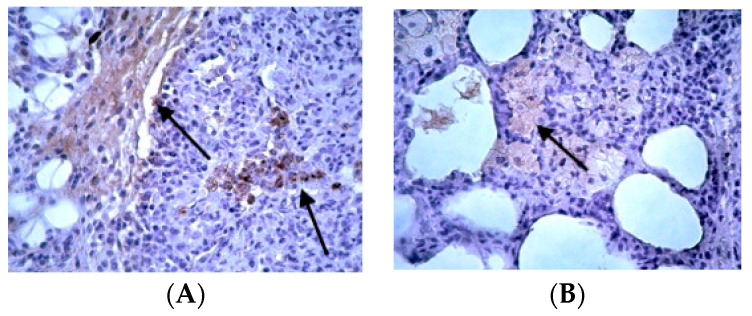
Representative data of MMP-9 expression: (**A**) BLM/SAL group, diffuse positive fibroblasts in the interstitial and alveolar epithelium (arrows); (**B**) BLM/EPO group, mild positive staining in alveolar epithelial cells (arrow).

**Figure 5:**
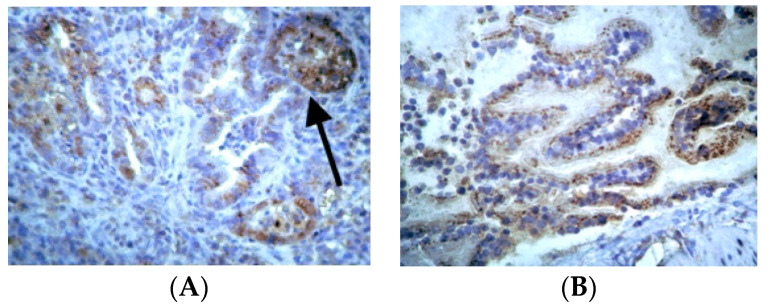
Representative data of EPO-R expression: (**A**) BLM/SAL group, multiple positive fibroblasts (arrow) flood the substrate and bronchial epithelium through freeing the lumen of the bronchi; (**B**) BLM/EPO group, mild positive staining in epithelial cells of the bronchus. No staining in the interstitial space.

**Figure 6:**
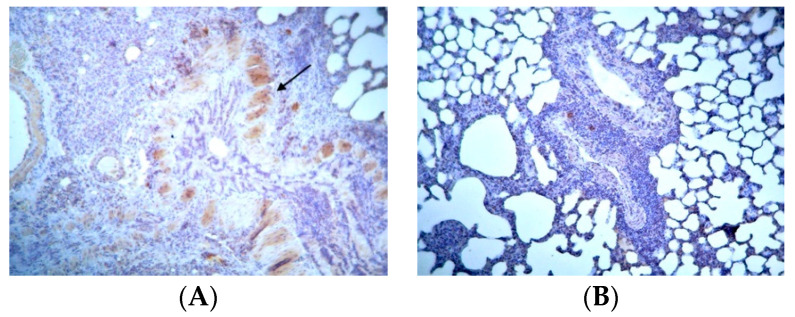
Representative data of Cyt-c expression: (**A**) BLM/SAL group, diffuse seizure of the mid-alveolar space by positive fibroblasts (arrow); (**B**) BLM/EPO group, absence of positive staining areas throughout the field of vision, healthy cells.

Protein expressions of COX-2, i-NOS, MMP-9, EPO-R, and Cyt-c were evaluated through immunohistochemical analyses. The expression was classified on a scale from 0 to 3. The expression of all five proteins was found to significantly differ among the groups. Specifically, the expression of all proteins was mostly classified as class 0 in control groups and the BLM/EPO group, while in the BLM/SAL group, their expression was mostly classified as class II. Immunohistochemistry results of the enzymes in the control groups (CNT, CNT SAL, CNT SAL/EPO), and percentages of expression for all groups are provided in the Supplementary Materials (Figures S1–S5). Representative enzyme expression data for the bleomycin groups (BLM/SAL, BLM/EPO) are shown in Figure 2, Figure 3, Figure 4, Figure 5 and Figure 6.

Concerning COX-2 expression, mild expression of COX-2 was observed in epithelial cells and smooth muscle tissue in control groups, while an absence of expression of COX-2 was observed in endothelium and substrate cells (Figure S1). In the BLM/SAL group, substrate areas with extensive fibrosis and positive pigmentation in the endoluminal bronchial wall were observed in most experimental animals. In the BLM/EPO group, maintenance of the median spatial and cellular structure and decreased expression of COX-2 were observed in the substrate, with fewer positive fibroblasts compared to the BLM/SAL group, along with a very limited number of positive bronchi, thus demonstrating a decrease in the degree of expression of COX-2 and the severe retreat of inflammation in animals treated with erythropoietin (Figure 2).

Concerning i-NOS expression, the expression of i-NOS was detected in a minimal number of cells on the substrate and in none in the smooth muscle fibers of the vessels in control groups (Figure S2). In the BLM/SAL group, staining was positive in areas of extensive fibrotic damage in most experimental animals. Positive cells appeared in the interstitial connective tissue and positive smooth muscle fibers in the bronchi and vessel membrane, indicative of the active fibrotic and oxidative process (Figure 3A). There was a very small number of fibroblasts on the substrate in the BLM/EPO group, and staining was only slightly positive in the medial fibrous muscle fibers, indicating that the oxidative stress has been seriously suppressed (Figure 3B).

Concerning MMP-9 expression, light expression of MMP-9 was observed in a small number of respiratory epithelial cells in control groups (Figure S3). Fibrosis was minimal or non-existent, and the cell’s architectural structure was normal. In the BLM/SAL group, staining was intense in the alveolar epithelium in almost all animals. Extensive fibrosis and positive cells were observed in both alveolar epithelium and interstitial space, indicating increased expression of MMP-9 in this group (Figure 4A). In the BLM/EPO group, mild expression of MMP-9 was observed. The fibrous areas were limited in size, and the respiratory epithelium almost improved as a whole (Figure 4B), indicating that apoptosis and inflammation had been significantly decreased.

Regarding EPO-R expression, mild expression of EPO-R was observed in the control groups (Figure S4). The staining was very weak in these animals, and no remarkable change in the cell membrane’s structural characteristics was observed where EPO-R was detected. In the BLM/SAL group, intense expression of the erythropoietin receptor was observed, with characteristic staining of the membrane of the substrate fibroblasts and the bronchial wall. Scattered macrophages and lymphocytes bear their color-intensive staining, indicating increased expression of EPO-R (Figure 5A). In the BLM/EPO group, EPO-R expression was also observed but reached a lower range than those of the previous group, indicating that apoptosis had been reduced (Figure 5B).

Concerning Cyt-C, its expression was mild and restricted to low levels in control groups (Figure S5). The staining was of low intensity in these animals, and small structural changes were observed in the alveolar space. In the BLM/SAL group, there was Cyt-C expression at very high levels, with diffuse lesions but also focal sites of staining in mid-alveolar fibroblasts, as well as in the outer vein of the vessels (Figure 6A). In the BLM/EPO group, staining—and, therefore, the protein’s expression—was at a lower level than in the previous group. A clear improvement was observed in the microscopic image, with a reduction in the number of positive fibroblasts in both the bronchial and alveolar space (Figure 6B), providing reassurance that apoptosis had been reduced again.

## 4. Discussion

Due to its ever-worsening course and fatal outcome, pulmonary fibrosis has been the subject of extensive scientific research focused on its etiology and pathogenesis, as well as diagnosis and treatment [25]. The present study provides evidence for the anti-inflammatory role of erythropoietin through COX-2 expression, its antioxidant role through i-NOS expression, its protective role through EPO-R expression, and its antiapoptotic role through MMP-9 and Cyt-C expression in an experimental bleomycin-induced rat model of IPF. Specifically, pulmonary fibrotic areas, as well as the intensity of fibrotic effects, such as the accumulation of lymphocytes and macrophages, were reduced in EPO-treated animals (Figure 1E). 

Furthermore, in all groups, body weight, hematocrit, and EPO serum levels were measured on the first and last day of the experiment. The weight of animals in which EPO was exogenously administered also presented a statistically significant increase, corresponding to the weight of control animals. Similar results have been reported by Ozer et al. in neonatal hyperoxia-exposed rats, where the body weights of the EPO-treated animals were not significantly different from those in the control group [26]. However, in a previous study, it has been reported that EPO decreases blood glucose levels in obese mice that are susceptible to diabetes and obesity through decreasing body weight and lowering hemoglobin A1c [27]. Overall, in our study, the administration of EPO resulted in increased or stable weight of treated animals compared to control animals, which likely reflects EPO’s protective effect on cellular metabolism and respiration. However, the underlying mechanism was not fully investigated. In particular, concerning the animals in the bleomycin group, their weight was decreased on the 14th day of the experiment. In previous studies, similar results have been observed, suggesting that the weight of bleomycin-treated animals decreased due to reduced food intake and hypoxia [27,28,29,30,31]. Furthermore, hypobaric hypoxia has been reported to cause weight loss, as higher metabolic rate and different energy output occur, together with decreased food intake and several endocrine factors, when examined at high altitude in obese human subjects [30].

Changes in hematocrit and EPO serum levels between the 1st and 14th days of the experiment were not significantly different. The findings of previous studies concerning hematocrit and EPO levels have been inconsistent [32,33,34]. It has been reported that hematocrit and EPO levels in different animal models either presented no significant differences between the 1st and 14th days, similar to our study [32], or were elevated on the 14th day of the experiment [33,34]. Concerning our results, the insignificant differences may be explained by the administered dose of EPO. Additionally, it has been suggested that low levels of EPO in serum may result from the influence of cytokines or other inflammatory mediators or from the great number of primitive erythroid progenitors [35]. In addition, researchers have suggested the important role of the endogenous erythropoietin system in the recruitment of endothelial progenitor cells in hypoxia-induced pulmonary hypertension mice, as well as in patients with acute myocardial infarction [36].

Furthermore, in our study, bleomycin-induced damage was confirmed through histochemical staining with hematoxylin–eosin, which demonstrated the severe damage caused by the fibrosis in the lung tissue. The severe damage included scars, diffuse inflammatory outbreaks, vast infiltration of the epithelium by macrophages and neutrophils, and erosion of the alveoli and bronchioles, all leading to lung reduction and destruction. These results were in accordance with previous reports that bleomycin led to a neutrophil-driven inflammatory response which subsequently transformed into a fibrotic response [37,38,39,40].

Regarding the extent of tissue injury due to fibrosis, as proven through the morphometric analysis, a statistically significant decrease in tissue injury was observed in animals treated with EPO. The pathological lesions that characterize pulmonary fibrosis appeared to be more limited and localized in EPO-treated animals compared to those with no treatment. Although limited data are available regarding the effects of EPO on bleomycin-induced fibrosis models, similar findings concerning its protective role on lung tissue injury have been reported in previous studies [26,28,41,42]. For example, Yoshimi et al. demonstrated that EPO reduced the histological degree of inflammation and fibrosis in bleomycin-induced pneumonitis in mice through the expression of phosphorylated Akt and Erk [28]. Moreover, in a study by Sigounas et al. using a bleomycin-induced fibrosis model, EPO was reported to ameliorate chemotherapy-induced fibrosis and endothelial damage [42].

In the present study, the expression levels of COX-2 i-NOS, MMP-9, EPO-R, and Cyt-C, examined using immunohistochemical methods, were chosen to assess the inflammatory and fibrotic processes in the experimental model of lung fibrosis. It is known that COX-2 and MMP-9 are expressed where uncontrolled degradation of the matrix takes place [43], while i-NOS is expressed in highly oxidizing environments with increased production of oxygen free radicals [44]. Moreover, EPO-R is upregulated in hypoxic and ischemic conditions, while Cyt-C is expressed in conditions of cell death and apoptosis [28].

More specifically, in our study, COX-2 was found to be highly increased in the bleomycin-induced fibrosis model animals. It has been shown, in several studies, that the expression of COX-2 is stronger under inflammatory conditions [45,46]. Furthermore, the increase in its expression has been observed not only in fibrosis and inflammation caused by bleomycin but also with other toxic substances such as silicone and losartan [46,47]. However, a limited number of specific studies have shown that overexpression of COX-2 in the lung leads to reduced fibroblast proliferation [48] and that COX-2-deficient mice are more susceptible to bleomycin-induced pulmonary fibrosis [49]. This inconsistency of findings might be due to the fact that the expression of COX2 has been investigated through a wide variety of COX-2 metabolites and mediators, considering diverse biological actions on different organs [50]. Herein, we further demonstrated the contribution of EPO to the reduction of COX-2. No previous studies have investigated EPO’s role in COX-2 expression to report similar results in in vivo models.

We also observed significantly decreased levels of i-NOS expression in EPO-treated animals. Indeed, i-NOS is expressed in highly oxidizing environments with increased production of oxygen free radicals and produces nitric oxide at a high flux rate in inflammatory and epithelial cells under conditions of inflammation [44]. Although there have been no reports in lung fibrosis models concerning the relationship between EPO and i-NOS expression, it has been demonstrated in several studies that the inhibition of i-NOS results in resistance to bleomycin-induced lung injury in mice [51,52]. Additionally, similar to our findings, other studies have shown the beneficial effects of EPO on cardiac function via the downregulation of i-NOS expression in rat [5] or mouse models [53]. Moreover, EPO’s protective role against necrotizing enterocolitis in newborn rats, through inhibiting the formation of nitric oxide, has been demonstrated [54]. Therefore, EPO may serve as an antioxidant agent in IPF treatment.

Furthermore, our results revealed increased MMP-9 expression in the bleomycin group, in accordance with previous reports in bleomycin models [55,56]. Our results also indicated that EPO inhibits the expression of MMP-9. Similar results have previously been reported by other researchers concerning EPO’s protective role against lung injury, via the inhibition of systemic and local expression of MMP-9, as well as TNF-alpha expression [12]. In a recent study, EPO was suggested to promote lung repair at the 14th day in hyperoxia-induced bronchopulmonary dysplasia injury in neonatal mice [57]. The mechanism investigated was mediated by inhibition of the TGF-β1 signaling pathway and the suppression of epithelial–mesenchymal transition, where protein expression of MMP-9 was decreased.

In addition, in the present study, EPO-R expression was found to be significantly decreased in EPO-treated animals when compared to the bleomycin group. It is known that EPO-R is rapidly expressed in hypoxia or ischemia, as observed in the bleomycin group in our study. Furthermore, our results of decreased EPO-R expression in EPO-treated animals revealed that EPO treatment protected the cells, and no EPO-R expression was required. Thus, a possible explanation for the increased expression of EPO-R during fibrosis or inflammation (BLM-animal group) is that more binding sites for endogenous EPO are available, allowing it to play its protective role. In the EPO animal group, these binding sites are covered by the exogenously administered EPO; therefore, there is no more need for high expression of EPO-R and endogenous EPO. Similar results have been previously revealed in a study of bleomycin-induced pneumonitis in mice, where EPO-R expression was found to be significantly decreased in bronchiolar epithelial cells, alveolar type II cells, and endothelial cells in EPO-treated animals, suggesting EPO’s protective role in these cells [28]. Similarly, in other studies, apoptosis was reduced in EPO-treated animals in a model of acute lung injury, as EPO-R expression and the proapoptotic Bcl-xL/Bax ratio were found to be significantly decreased [41]. This antiapoptotic activity of EPO might suggest a promising role in IPF treatment. Furthermore, in a recent study, it was reported that normal erythropoietin (EPO)/EpoR signaling in renal tubules provides defense against renal tubular injury through maintenance of the autophagy–apoptosis balance and peritubular capillary integrity [58]. Furthermore, it has not yet been clearly established whether EPO’s protective role is mediated by EPO-R alone or in combination with the EPO beta common receptor (βcR) [53].

Cyt-C is a proapoptotic molecule which is released by the mitochondria when changes in mitochondrial permeability occur, leading to cytosolic and nuclear apoptosis [59]. Our results are in accordance with previous studies, where increased Cyt-C levels have been associated with lung inflammation and fibrosis [59,60,61]. In our study, Cyt-C expression was found to be significantly decreased in EPO-treated animals when compared to those in the bleomycin group. There have been no previous reports on the role of EPO in association with Cyt-C expression in lung fibrosis models. However, EPO’s beneficial effects in relation to Cyt-C expression have been investigated in other systems. More specifically, EPO has been shown to decrease Cyt-C expression, playing a protective role against ischemia [62]. Previously reported results concerning skeletal muscles are inconsistent, as EPO’s effect has shown to either increase [63] or decrease [64] Cyt-C expression. While EPO’s antiapoptotic role was revealed in our study, taking into consideration the heterogeneity of Cyt-C expression in different tissues, the relationship between EPO and Cyt-C remains to be further elucidated.

There are some limitations to this study. Quantitative assessment of the proteins that had been previously studied and the investigation of additional time points would allow better understanding of the effects of EPO in animal bleomycin models. Furthermore, investigating the roles of other molecules could reveal further biological mechanisms, as both innate and adaptive inflammatory processes are involved in the pathogenesis of IPF (5).

## 5. Conclusions

In summary, the present study aimed to explore new therapeutic methods for pulmonary fibrosis, and, in particular, it examined the possible anti-inflammatory, antioxidant and antiapoptotic properties of erythropoietin in the lungs of fibrotic rats induced through the exogenous administration of bleomycin, based on the experimental bleomycin model applied. This is the first study to demonstrate that erythropoietin impeded pulmonary fibrosis by improving the extent of tissue damage and reducing oxidative load and apoptosis. Our results may constitute a starting point for more research on the impact of erythropoietin on pulmonary fibrosis.

## Figures and Tables

**Table 1:** Results of body weight, hematocrit, and erythropoietin for all experimental groups at day 1 and day 14 (mean ± SD).

	Body Weight (g)		Hematocrit (%)		Erythropoietin (pg/mL)	
Group	Day 1	Day 14	Day 1	Day 14	Day 1	Day 14
CNT	260.4 ± 12.5 *	325.2 ± 14.4 *	48.2 ± 1.3	47.8 ± 1.3	5.9 ± 0.2	5.8 ± 0.2
CNT SAL	273 ± 46.8 *	321.2 ± 43.2 *	48 ± 0.7	48.6 ± 0.8	6.1 ± 0.5	5.7 ± 0.5
CNT SAL/EPO	268 ± 47.2 *	301 ± 41.2 *	47.4 ± 0.8	48.2 ± 1.3	6.3 ± 0.3	5.7 ± 0.6
BLM/SAL	259.3 ± 23.5 *	227.2 ± 23 *	48.3 ± 1.1	48.6 ± 0.7	5.9 ± 0.4	5.1 ± 0.9
BLM/EPO	263.7 ± 46.1 *	289.2 ± 51.8 *	48.5 ± 1	49.1 ± 1.1	6.4 ± 0.4	6 ± 0.7

CNT: no formulation, CNT SAL: saline, CNT SAL/EPO: saline and EPO, BLM/SAL: bleomycin, BLM/EPO: bleomycin and EPO, * *p* < 0.001 with statistically significant differences between days 1 and 14.

## Data Availability

The original contributions presented in the study are included in the article/Supplementary Material, further inquiries can be directed to the corresponding author.

## Supplementary Materials

The following supporting information can be downloaded at: https://www.mdpi.com/article/10.3390/jpm14090972/s1, Figure S1. COX-2 expression in experimental models; Figure S2. I-NOS expression in experimental models; Figure S3. MMP-9 expression in experimental models; Figure S4. EPO-R expression in experimental models; Figure S5. Cyt-c expression in experimental models.
