# Supplementary material for: Erythropoietin Reduces Inflammation, Oxidative Stress, and Apoptosis in a Rat Model of Bleomycin-Induced Idiopathic Pulmonary Fibrosis
Source: J Pers Med. 2024 Sep 13;14(9):972. doi: 10.3390/jpm14090972 (PMC11433300; doi:10.3390/jpm14090972)
Supplement: Supplementary file 1 [file jpm-14-00972-s001.zip › jpm-3150759-supplementary.pdf]

Journal of
Personalized
Medicine

MDPI

Supplementary Materials:

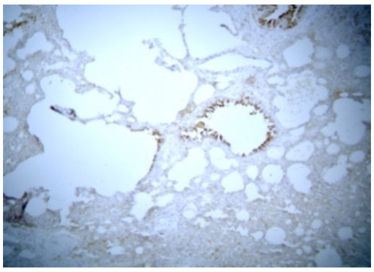

A (CNT)

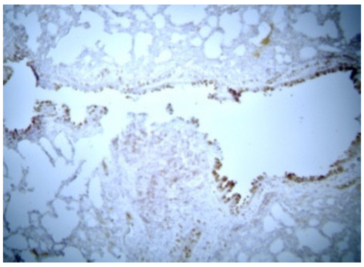

B (CNT SAL)

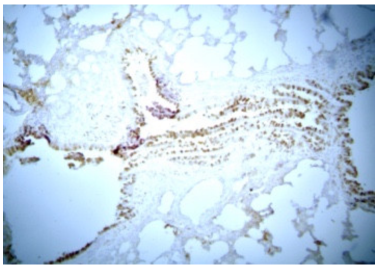

C (CNT SAL/EPO)

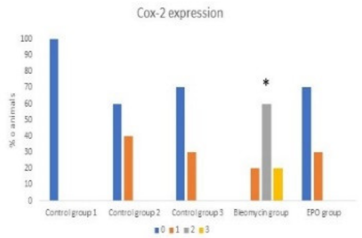

D (Cox-2 expression)

Figure S1. COX-2 expression in experimental models. A) Mild expression of Cox-2 in the epithelial cells. B) Minimal expression of the enzyme in the cells of bronchus. C) Intense staining of smooth muscle fibers. D) Cox-2 expression as a percentage (%) of the experimental animals per study group classified on a 0-3 scale. * p<0.001, with statistically significant differences between BLM/SAL and BLM/EPO group and BLM/EPO and control groups.

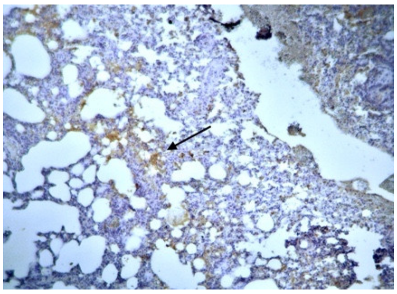

A (CNT group)

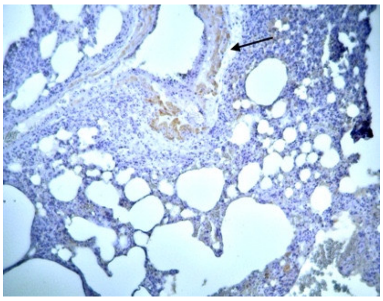

B (CNT SAL)

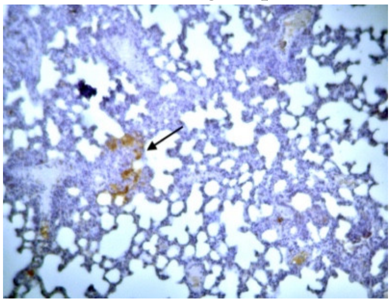

C (CNT SAL/EPO)

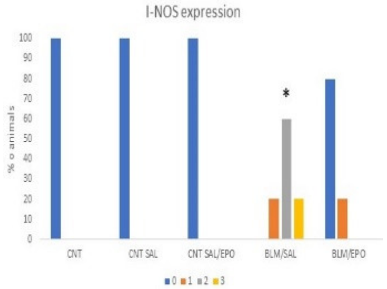

D (I-NOS expression)

Figure S2. I-NOS expression in experimental models. A) Few positive cells in the respiratory epithelium (arrow). B) Minimal staining in the bronchial cells of the substrate (arrow). C) Physiological cell architecture and parenchyma with minimal positive cells on the substrate (arrow). D) I-NOS expression as a percentage (%) of the experimental animals per study group classified on a 0-3 scale. * p<0.001, with statistically significant differences between BLM/SAL and BLM/EPO group and BLM/EPO and control groups.

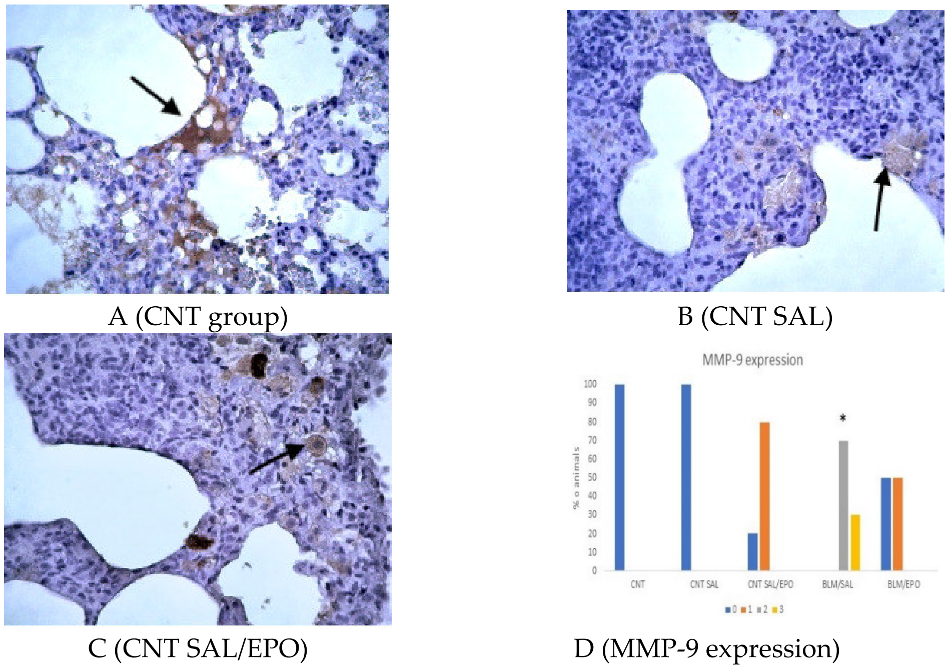

Figure S3. MMP-9 expression in experimental models. A) Minimum positive staining in the interstitial space (arrow). B) Mild pneumonitis with minimal positive staining in the interstitial space and not in the alveolar epithelium (arrow). C) Minimum staining only in substrate cells. Positive fibroblast. (arrow). D) MMP-9 expression as a percentage (%) of the experimental animals per study group classified on a 0-3 scale. * p<0.001, with statistically significant differences between BLM/SAL and BLM/EPO group and BLM/EPO and control groups.

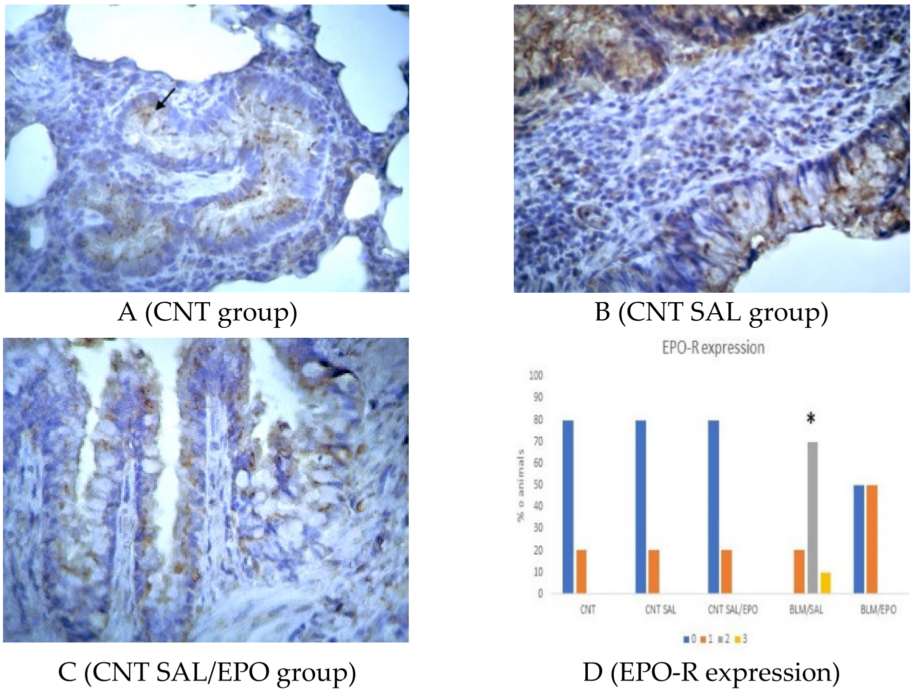

Figure S4. EPO-R expression in experimental models. A) Minimum staining of fibroblasts (arrow). B) Mild positive staining due to pneumonitis. Presence of positive fibroblasts in the interstitial space. EPO-R expression as a percentage (%) of the experimental animals per study group classified on a 0-3 scale. * p<0.001, with statistically significant differences between BLM/SAL and BLM/EPO group and BLM/EPO and control groups.

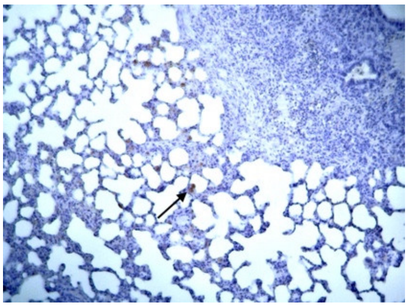

A (CNT group)

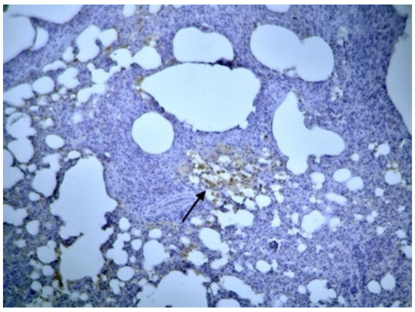

B (CNT SAL group)

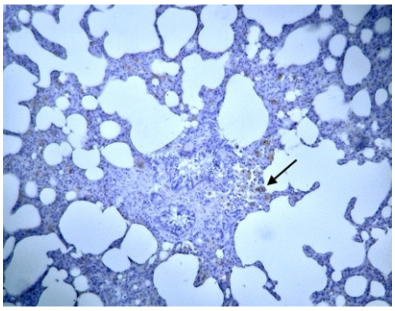

C (CNT SAL/EPO group)

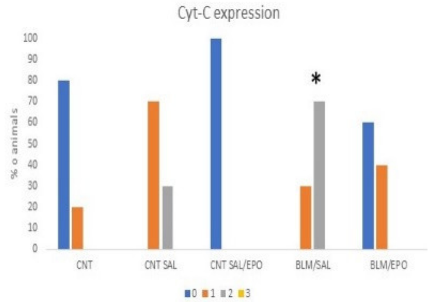

D (Cyt-c expression)

Figure S5. Cyt-c expression in experimental models. A) Positive fibroblasts in the alveolar area (arrow). B) Limited incidence of positive fibroblasts in the mid-esophagus of group 5 (arrow). C) Stronger presence of positive fibroblasts in group 2 than in groups 1 and 5 (arrow). D) Diffuse seizure of the mid-alveolar space by positive fibroblasts (arrow). E) Absence of positive staining areas throughout the field of vision, healthy cells. F) Cyt-c expression as a percentage (%) of the experimental animals per study group classified on a 0-3 scale. * p<0.001, with statistically significant differences between BLM/SAL and BLM/EPO group and BLM/EPO and control groups.
